# The Role of Multimodality Imaging in Cardiomyopathy

**DOI:** 10.1007/s11886-024-02068-9

**Published:** 2024-05-16

**Authors:** Jonathan A. Pan, Amit R. Patel

**Affiliations:** Cardiovascular Division, Department of Medicine, University of Virginia Health System, 1215 Lee Street, Box 800158, Charlottesville, VA 22908 USA

**Keywords:** Cardiomyopathy, Multimodality, Echocardiography, Magnetic Resonance, Nuclear Imaging, Computed Tomography

## Abstract

**Purpose of Review:**

There has been increasing use of multimodality imaging in the evaluation of cardiomyopathies.

**Recent Findings:**

Echocardiography, cardiac magnetic resonance (CMR), cardiac nuclear imaging, and cardiac computed tomography (CCT) play an important role in the diagnosis, risk stratification, and management of patients with cardiomyopathies.

**Summary:**

Echocardiography is essential in the initial assessment of suspected cardiomyopathy, but a multimodality approach can improve diagnostics and management. CMR allows for accurate measurement of volumes and function, and can easily detect unique pathologic structures. In addition, contrast imaging and parametric mapping enable the characterization of tissue features such as scar, edema, infiltration, and deposition. In non-ischemic cardiomyopathies, metabolic and molecular nuclear imaging is used to diagnose rare but life-threatening conditions such amyloidosis and sarcoidosis. There is an expanding use of CCT for planning electrophysiology procedures such as cardioversion, ablations, and device placement. Furthermore, CCT can evaluate for complications associated with advanced heart failure therapies such as cardiac transplant and mechanical support devices. Innovations in multimodality cardiac imaging should lead to increased volumes and better outcomes.

## Introduction

The prevalence of heart failure (HF) continues to grow globally with increasing financial burden worldwide. In 2017, there were 64.3 million people with HF globally [1] and 1.2 million HF admissions in the United States (US) alone [2]. The annual cost of HF per patient is $28,950 in the US [3] and the lifetime costs of HF is $126,819 per patient internationally [4]. Interestingly, there has been an increase in prevalence in HF globally, especially those with preserved ejection fraction, but a decline in incidence in the last 10 years [5]. This trend likely reflects a summation of our aging population, an emphasis on early diagnosis, and improvement in treatment options.

Cardiac imaging is an essential step in the initial evaluation of patients with suspected HF for diagnosis and prognostication [6]. Identifying the cause of an underlying cardiomyopathy is necessary for guiding disease-specific therapies, predicting adverse events, and determining subsequent testing and appropriate monitoring. Because of its versatility and availability, echocardiography is the first line imaging modality for characterizing new cardiomyopathies or evaluating for clinical changes in those with known diagnosis. However, echocardiography is heavily dependent on image quality, operator experience, and interobserver variability among readers. Therefore, the 2022 Heart Failure Guidelines recommends that when echocardiography is inadequate, alternative imaging such as cardiac magnetic resonance (CMR), cardiac computed tomography (CCT) or radionucleotide imaging should be used for assessment of left ventricular ejection fraction (LVEF) [6].

According to Medicare data from 2010–2019, CMR, CCT, and positron emission tomography (PET) have doubled in volume while echocardiography has remained steady year to year [7]. Increasing familiarity among providers, incorporation into new guidelines, and advances in technology has accelerated the use of multimodality imaging in the diagnosis and management of cardiomyopathies. Furthermore, advanced imaging has enriched our understanding of the mechanisms driving HF, aided in the development of targeted therapies, and bolstered our appreciation of the vast number of phenotypes that make up the clinical syndrome. The goal of this paper is to provide a practical overview of advanced cardiac imaging modalities, review appropriate uses in non-ischemic cardiomyopathy (NICM), and highlight promising new applications.

## Echocardiography

In patients with suspected cardiomyopathy, echocardiography continues to be the first test of choice [8]. With echocardiography, a clinician can measure left ventricular (LV) and right ventricular (RV) chamber sizes, wall thickness, and systolic function. In addition, they can visualize regional wall motion abnormalities, valvular disease, and congenital disorders. Color Doppler and spectral Doppler are essential tools for identifying areas of flow acceleration, quantify valvular disease, and grade diastolic function. Strain with speckle tracking has found increasing use in clinical practice, especially in the diagnosis of early systolic heart failure, hypertrophic cardiomyopathy, and cardiac amyloidosis [9]. With improvements in computing power and innovations in transducer technology, three-dimensional (3D) echocardiography has become widely available, allowing for more accurate and reproducible measurements of ejection fraction and volume [10]. With this one modality, there are numerous parameters that can be used to assess for a variety of cardiomyopathies, and the amount of content is beyond this scope of this review.

Although echocardiography is a highly utilized modality, clinicians should recognize that it is a resource intensive service that requires specialized sonographers, equipment, software, and clinical training. As a result, there have been initiatives to improve the appropriate use of echocardiography [11, 12] and its availability. Point of care ultrasound (POCUS) is increasingly being used by both cardiology and non-cardiology clinicians. In a study with 250 patients referred for standard echocardiography [13], they compared POCUS to physical exams for detecting suspected cardiac conditions. POCUS identified 82% of patients with abnormal echocardiogram, had a significantly better diagnostic accuracy than physical exam (71% vs 31%, p < 0.001), and was associated with lower downstream costs. Artificial intelligence (AI) guided echocardiography is also being developed to assist sonographers and cardiologists [14]. There are several AI studies demonstrating high accuracy for identifying standard echocardiography views [15], measuring LV ejection fraction [16], and differentiating between cardiomyopathies [17]. Therefore, technological advances in echocardiography will continue to play a crucial role in diagnosis and management of cardiomyopathies.

## Cardiac Magnetic Resonance

For newly diagnosed cardiomyopathies, CMR offers several tools for narrowing the differential diagnosis and subsequent risk stratification. CMR allows for accurate measurements of systolic function and chamber sizes. In addition, it can better visualize myocardial segments and valves that are not easily acquired by traditional echocardiography views. Because fewer assumptions are required, CMR is considered the reference standard for volumetric quantification [18]. CMR can also characterize myocardial tissue with late gadolinium enhancement (LGE) and quantitative parametric mapping based on T1 and T2 recovery. Therefore, CMR is an effective modality for guiding downstream testing and treatment.

### Volumetric Quantification

CMR ventricular measurements are made from a short-axis stack that covers the entire ventricle. The LV and RV ejection fraction and volume are calculated using the Simpson’s summation of discs method. The myocardium and cavity of each short axis slice is contoured to create a stack of disks that accurately reflect the myocardial structure. This differs from 2D echocardiography, which uses the Simpson’s biplane method with the 2-chamber and 4-chamber long-axis views to make assumptions about the shape of the disks and therefore an estimate of the volume. As a result, the LVEF can often vary between the modalities. CMR offers high reproducibility for both intra- and inter-observer comparisons. In lower LVEF ranges, 2-dimensional (2D) echocardiography often overestimates LVEF compared to CMR [19, 20•, 21]. In a study evaluating the impact of CMR on implantable cardioverter-defibrillators (ICD), CMR reclassified 41% of patients with LVEF between 25–40% by echocardiography [19]. Additionally, LVEF by CMR has been shown to be a better predictor of mortality in patient referred for primary prevention ICD when compared to echocardiography [20•].

### Morphology Definition

**Fig. 1:**
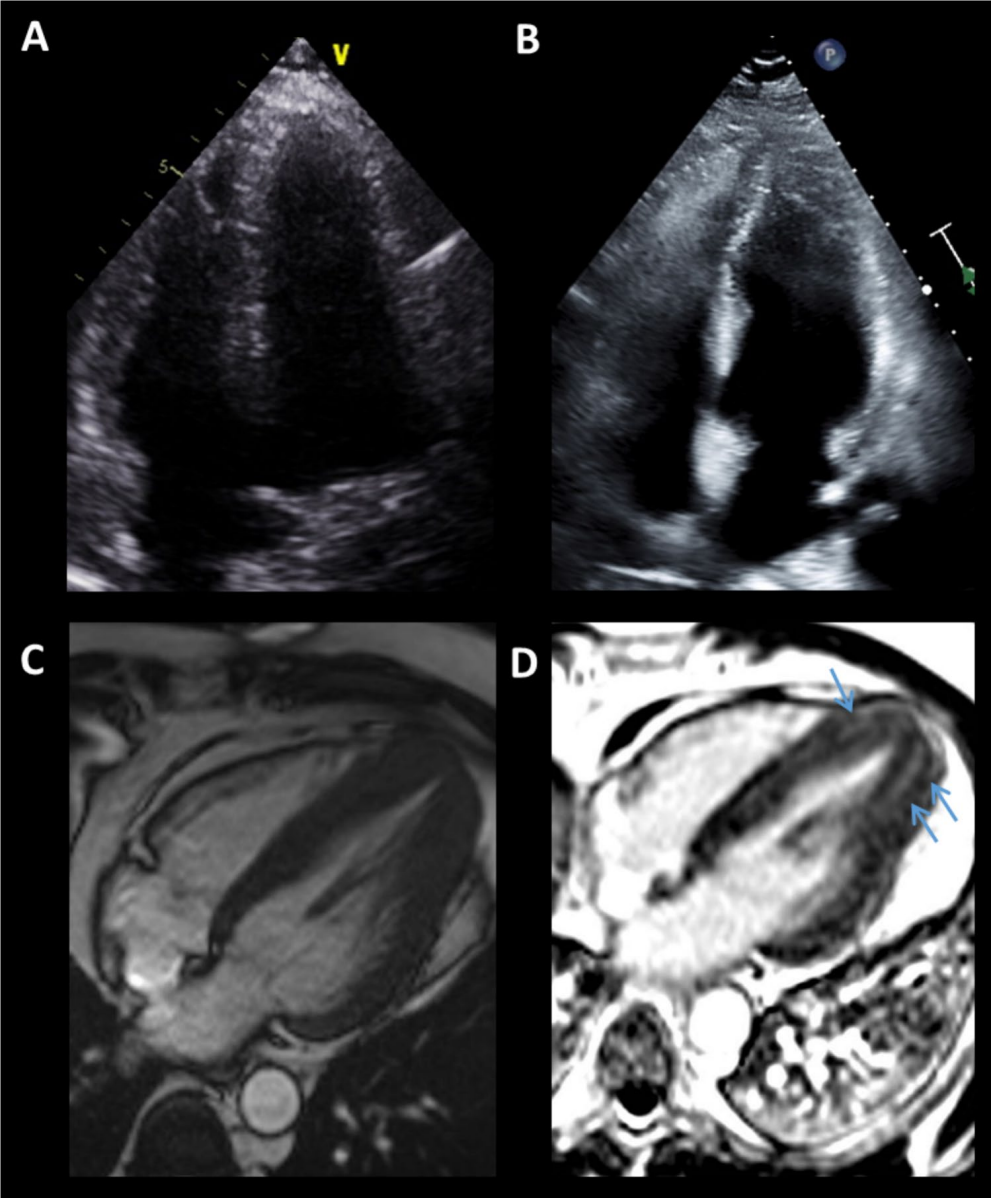
Apical Hypertrophic Cardiomyopathy. Apical hypertrophic cardiomyopathy was not seen on the initial echocardiography (**A**) but later diagnosed on a repeat study a year later (**B**). Cardiac magnetic resonance imaging confirmed the diagnosis by demonstrating the “spade-like” apex on cine (**C**) and patchy fibrosis in the hypertrophied segments (*blue arrows*) on late gadolinium enhancement imaging (**D**)

Cine CMR can acquire cardiac views with high spatial and temporal resolution, which can characterize morphologies that may be unique or of particular importance to specific cardiomyopathies. Hypertrophic cardiomyopathy (HCM) is the most common genetic cardiomyopathy occurring in 1 in 200 to 500 individuals [22]. Sarcomere gene mutations lead to hypertrophy and replacement fibrosis, presenting with heterogeneous phenotypes and variable expression. HCM is diagnosed in adults based on a maximum LV thickness ≥ 15 mm, with a lower cut-off of 13-14 mm in those with family history of HCM or positive genetic testing [23]. The risk of sudden cardiac death (SCD) is correlated with the maximum wall thickness, with 20-year cumulative risk of nearly zero for patients with wall thickness ≤ 19 mm and 40% for those with ≥ 30 mm [24]. There are multiple phenotypic variants of HCM based on location of hypertrophy, septal curvature, and the level of blood flow obstruction if present. Apical HCM is an important variant that is a more common cause of HCM in Asian populations and results in a “spade-like” appearance in the LV cavity (Fig. 1). Apical aneurysms can develop presumably from repetitive wall stress and impaired perfusion leading to subsequent scarring [25]. The aneurysm has a particularly high risk of thromboembolic event, arrhythmias including ventricular tachycardia (VT) and HF. HCM related deaths are threefold greater in patients with apical aneurysms when compared to those without aneurysms [26]. However, around 40% of apical aneurysms are missed by echocardiography but may be identified on CMR [25]. Other morphologic abnormalities such as myocardial crypts, systolic motion of the mitral valve, elongated mitral leaflets, apically displaced papillary muscles, and right ventricular hypertrophy can also be seen with HCM. These findings can help differentiate HCM from other mimics such as glycogen/lysosomal storage diseases, cardiac amyloidosis, or hypertensive heart disease.

Left ventricular non-compaction cardiomyopathy (LVNC) is also easily identified by CMR based on the prominent trabeculae and deep recesses in non-compacted myocardium. LVNC is due to failure of the embryological spongy myocardium to compact after coronary vasculature development [27]. LVNC is a rare cause of HF in adults, but is present in 9.2% of children with a primary cardiomyopathy, placing itself as the third most common cardiomyopathy in the pediatric population after HCM and dilated cardiomyopathy [28]. Diagnosis can be challenging as prominent myocardial trabeculations can be seen in other cardiomyopathies as well as healthy patients. Patient who carry the diagnosis of LVNC tend to have more segmental trabeculations in the inferior, lateral, and apical areas [29]. The most commonly accepted criteria for diagnosis is a ratio > 2.3 for the thickness of noncompacted to compacted myocardium measured at end-diastole by CMR, which has a reported sensitivity and specificity of 86% and 99% [30]. Another proposed method is to measure total non-compacted myocardial mass index and percentage, which has a higher diagnostic accuracy [31]. However, limitations of all the diagnostic criteria for LVNC are well documented [32]. The most common complications of LVNC are HF, ventricular arrhythmias, and thromboembolic events, with the latter being due to stasis of blood in the recesses between the trabeculae. In a study following 106 patients with LVNC, 26% patients died or underwent heart transplant over a 2.9 year follow-up period [33].

Arrhythmogenic cardiomyopathy (ACM) is another diagnosis for which CMR is the modality of choice. ACM was originally known as arrhythmogenic right ventricular cardiomyopathy given that fibro-fatty replacement was primarily occurring in the RV [34]. This contributes to RV dysfunction and ventricular arrhythmias. However, there are other variants with either left ventricular or biventricular involvement [35]. ACM accounts for 10% of unexpected sudden cardiac death based on autopsies, with one third of cases occurring in the fourth decade of life [36]. ACM is diagnosed based on the 2010 Task Force criteria, which incorporates imaging, histology, EKG abnormalities, arrhythmias, and family history [37]. The imaging component requires evidence of RV regional wall motion abnormalities or dyssynchrony and either reduced RV ejection fraction or dilated end-diastolic cavity size [37]. However, these findings can be subtle and difficult to acquire with echocardiography due to complex structure of the RV. As a result, CMR is heavily used to obtain accurate volumetric measurements and evaluate the RV free wall and outflow tract. The proposed 2020 international criteria also include findings for LV phenotypes [38]. CMR is additionally very helpful to differentiate ACM from mimics such as the cardiac sarcoidosis, congenital heart disease, and normal variants such as the “butterfly apex.” This is a normal anatomic variation in which the LV and RV have separate apices in the shape of a butterfly. However, the separate RV apex is often misdiagnosed as an aneurysmal or dyskinetic segment [39].

### Focal Scar Patterns

One of the strengths of contrast CMR is its ability to identify scar in the myocardium. LGE imaging specifically enhances areas of retained gadolinium contrast in the myocardium, which is due to expanded extracellular space from myocyte loss and replacement fibrosis [40]. There is unequivocal evidence that the presence of scar detected using LGE is associated with an increased risk of all-cause mortality, HF hospitalizations, and SCD in patients with NICM [41]. A higher LGE burden is also associated with worse outcomes. In patients with HCM, LGE that is greater than 15% of the LV mass had a threefold increase in SCD and ICD discharge [42]. Although there is no consensus of the best quantification method for LGE in HCM, it is still considered by experts to be a powerful risk stratification tool for SCD. Given the prevalence of scar in akinetic and aneurysmal segments, LGE is also useful for both detecting LV thrombus and identifying patients at risk of subsequent embolic events [43].

**Fig. 2:**
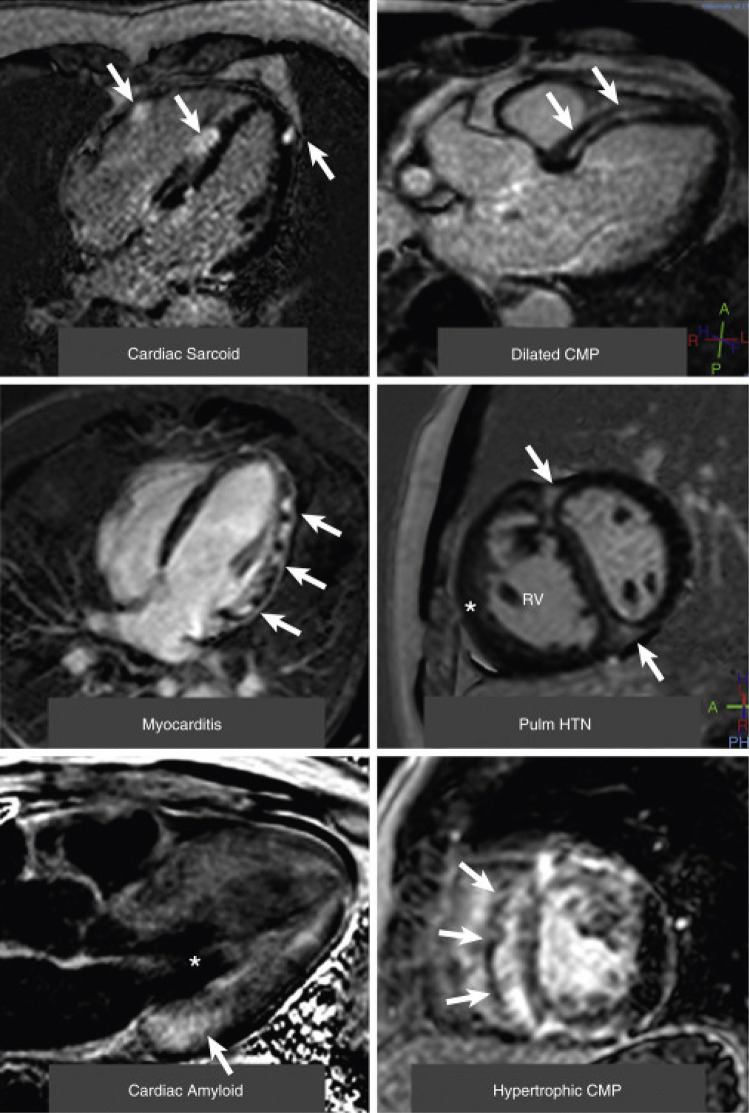
Examples of Late Gadolinium Enhancement in a Variety of Nonischemic Cardiomyopathies. (Top left) A 4-chamber view of patchy distribution of late midwall and epicardial late gadolinium enhancement (LGE) (*arrows*) in cardiac sarcoidosis. (Top right) A 3-chamber view of a midwall stripe pattern of LGE (*arrows*) in dilated cardiomyopathy (CMP). (Middle left) A 4-chamber view of patchy epicardial and midwall LGE along the lateral wall (*arrows*) in myocarditis. (Middle right) A midventricular short-axis image of LGE in the anterior and inferior right ventricular insertion points (*arrows*) in pulmonary hypertension (Pulm HTN) with right ventricular (RV) hypertrophy (*). (Bottom left) A 3-chamber view of a LGE image in cardiac amyloidosis. The left ventricular blood pool is nulled (*), and there is subtle circumferential subendocardial LGE throughout the left ventricle. The LGE is most pronounced at the base of the left ventricle within hypertrophied myocardium (*arrow*). (Bottom right) A midventricular short-axis image in a patient with hypertrophic cardiomyopathy with evidence of asymmetrical septal hypertrophy with extensive midwall LGE within the hypertrophied myocardium (*arrows*). Adapted from Patel et al. JACC Cardiovasc Imaging, 2017. 10(10 Pt A):1180–1193, with permission from Elsevier [44]

The pattern for LGE can be used to distinguish between types of cardiomyopathies (Fig. 2) [44]. For example, the location of LGE within the myocardial wall can easily differentiate between ischemic and non-ischemic cardiomyopathy. In the former, ischemia occurs first in the subendocardium due to reduced perfusion pressure from epicardial coronary disease. As a result, LGE is seen in the subendocardium of a diseased coronary territory and can extend to the epicardium during myocardial infarction. Transmural involvement of the coronary territory suggests poor viability and decreased likelihood of recovery of function after revascularization [45]. In dilated cardiomyopathy, a little less than a third of patients will have mid-wall stripe LGE in the interventricular septum [46], for which the presence and extent is an independent predictor SCD, HF hospitalization, transplant, and death [47]. Sarcoidosis is an inflammatory disorder with multiorgan involvement of noncaseating granulomas. LGE can be seen in commonly involved segments such as the LV basal septum and lateral wall in the epicardium and mid-myocardium. The presence of LGE is associated with a 3.5-fold increase in annualized mortality rate [48]. Cardiac amyloidosis is an infiltrative disorder in which proteins such as immunoglobulin light chains and transthyretin, are deposited in the myocardium and result in expansion of the extracellular space. The 2 most common types are light chain (AL) amyloidosis from plasma cell-dyscrasias or transthyretin amyloidosis (ATTR) from misfolded albumin produced by the liver. LGE is commonly seen as diffuse subendocardial involvement of the base and middle of the left ventricle [49]. In late-stage cardiac amyloidosis, the degree of LGE can be so diffuse that the images are difficult to interpret due to alterations in the inversion time, which is considered pathognomonic the cardiac amyloidosis and a strong predictor of mortality [50]. In chronic Chagas disease, LGE is seen in the apex and inferolateral wall that can be either focal, transmural, or diffuse in extent [51]. RV insertion point LGE is a common but nonspecific finding in patients in patients with HCM and pulmonary hypertension [52, 53].

### Parametric Mapping

**Fig. 3:**
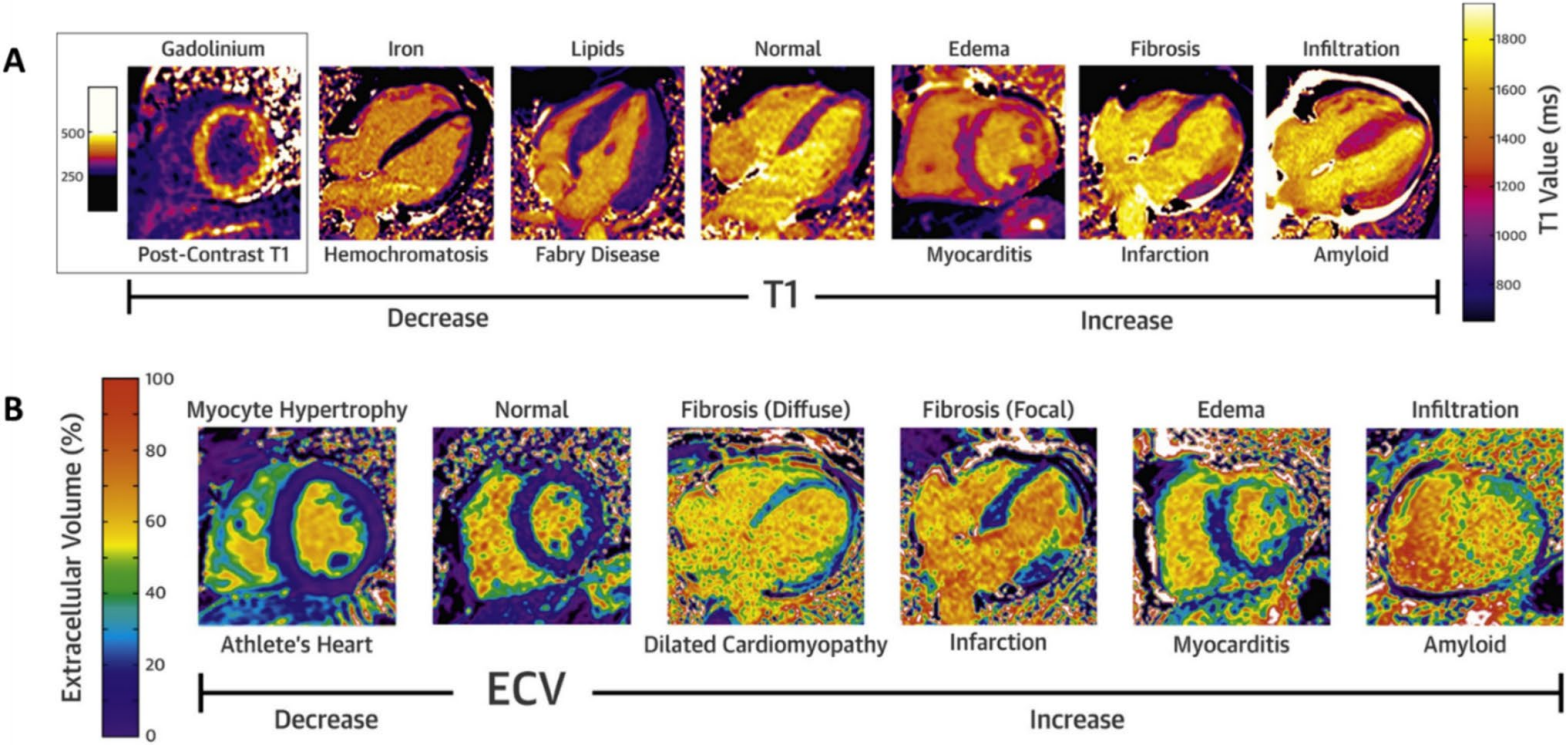
Native T1 and ECV Mapping in Different Cardiomyopathies.In native T1, most cardiomyopathies cause increase in values except for iron deposition and Fabry disease (**A**). For extracellular volume mapping (ECV), the percentage is elevated with fibrosis, infiltration, and edema (**B**). Adapted from Robinson et al. JACC Cardiovasc Imaging, 2019. 12(11 Pt 2):2332–2344, with permission from Elsevier [54]

CMR offers a quantitative method to characterize myocardial tissue using parametric mapping. By measuring the magnetic relaxation times in the longitudinal (T1) and transverse (T2) directions to the static magnetic fields from the scanner, color-encoded maps can be constructed for which each pixel represents a T1 or T2 value (Fig. 3) [54]. This allows the reader to visualize and measure the global and regional T1 and T2 values [55]. T2 elevation is generally considered to be more specific to myocardial edema. In canine models that underwent myocardial infarction, T2 values were shown to strongly correlate with percent water content in the infarct territories and therefore to be an excellent marker of edema [56]. Native T1 values are elevated in any disease process that alters intracellular and extracellular content such as edema, fibrosis, and necrosis [57, 58]. After the administration of gadolinium contrast, T1 relaxation times will shorten predominantly based on volume of distribution of contrast in the extracellular space [59]. Extracellular volume (ECV) maps can be calculated based on the pre- and post-T1 maps of the myocardium and blood pool and then adjusted for the hematocrit [60]. Conceptually, ECV mapping represents the change in tissue T1 compared to plasma T1 with gadolinium contrast and is therefore an indirect measurement interstitial volume fraction of the myocardium. Elevated ECV can reflect expansion of the extracellular space due to diffuse fibrosis [61] or infiltrative processes [62]. A synthetic ECV can be calculated without blood sampling based the T1 of the blood pool, which has been validated in multiple cohorts [63, 64] but can be less accurate in extremes of hematocrits [65]. Although ECV can vary depending on field strength, T1 mapping sequence, and MRI vendor, the normal range is generally more consistent and reproducible than that of T1 and T2 mapping. Additionally, ECV values are reported as fractions, as opposed to millisecond units used for T1 and T2 values.

Parametric mapping sequences are crucial for evaluating inflammation of the myocardium [66]. The Lake Louise Criteria II requires both T1- and T2-based imaging to diagnose acute myocarditis [67]. In the MyoRacer-Trial, patients underwent endomyocardial biopsy and CMR imaging with T1 and T2 mapping for acute and chronic symptoms from suspected myocarditis. T1 and T2 mapping had a diagnostic accuracy of 81% and 80% for acute myocarditis. Interestingly, only T2 mapping was able to diagnose chronic myocarditis with an accuracy of 73%. COVID-19 related myocarditis has also been increasingly recognized by CMR. In a study by Puntmann et al. [68], a total of 100 patients who recovered from mild to moderate COVID-19 underwent CMR 2 to 3 month after their initial COVID test. Surprisingly, 78 of the patients had abnormal CMR findings, which included reduced biventricular function and elevated T1 and T2 times; however, the study has been criticized for the lack of control subjects who did not have COVID-19 [69]. Immune checkpoint inhibitor (ICI) myocarditis is a well-known but rare complication of the immunotherapy, with a reported fatality rate of up to 40% [70]. A large multicenter registry of 136 patients with biopsy proven ICI myocarditis found abnormal T1 and T2 values in 78% and 43% respectively. Furthermore, only native T1 was independently associated with MACE. These findings suggest that there is more myocardial injury than the extent of edema detected using T2 imaging that occurs in ICI myocarditis.

Parametric mapping with CMR is also useful for diagnosing infiltrative diseases. Fabry disease (FD) is rare X-linked lysosomal storage disease that results in the accumulation of glycosphingolipids throughout the body. Its accumulation in the myocardium results in asymmetric septal hypertrophy and eventual systolic dysfunction with inferolateral wall thinning [71]. In early stages of Fabry disease, reduction of native T1 due to glycosphingolipids storage has a high specificity of up to 99% [72]. As the disease progresses, the glycosphingolipids accumulation lead to replacement fibrosis and pseudonormalization of the native T1 [72]. Similar to LGE and focal fibrosis, ECV and diffuse fibrosis provide important diagnostic and prognostic information. In a prospective study by Cadour et al. [73••], 225 patients with non-ischemic dilated cardiomyopathy underwent CMR and were followed for 2 years. They showed that ECV was independent predictor of HF and arrhythmia related events [73••]. In cardiac amyloidosis, pooled analyses have shown that ECV has both a higher diagnostic odds ratio and mortality hazard ratio than LGE [74]. Because the condition is entirely driven by the deposition of amyloid proteins in the extracellular space, cardiac amyloidosis often has the highest ECV of all NICM, nearing levels seen in infarcted myocardium [75]. CMR can also detect iron overload in cardiac siderosis, which occurs in transfusion-dependent anemias or hemochromatosis [76]. T2* relaxation is the decay of transverse magnetization (T2) in the presence of magnetic field inhomogeneity, which can be induced by iron deposition. T2* relaxation time decreases linearly with increasing iron load and predict the development of ventricular dysfunction [77].

## Cardiac Nuclear Imaging

Advanced nuclear imaging employs radionucleotides with unique biodistribution and tissue targeting properties. Single-photon emission computed tomography (SPECT) and positron emission tomography (PET) are commonly used to identify perfusion defects in ischemic cardiomyopathy. However, nuclear medicine also plays an important role in the evaluation of cardiac amyloidosis and sarcoidosis, and is necessary for guiding management.

### Molecular Imaging

**Fig. 4:**
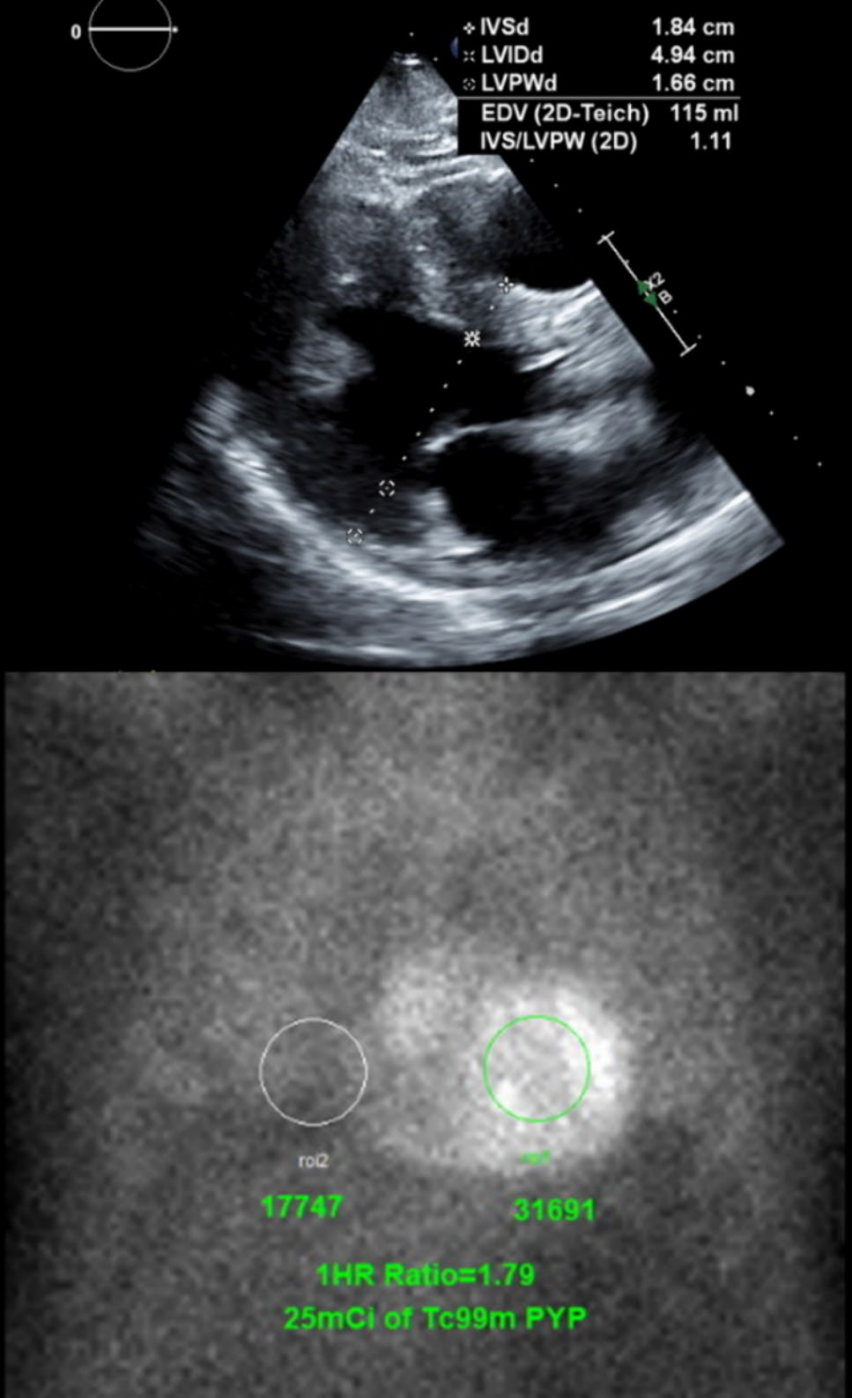
Suspected Transthyretin Cardiac Amyloidosis Diagnosed by ^99m^Tc-pyrophosphate Scintigraphy. (Top) Patient with new heart failure and severe concentric hypertrophy parasternal long-axis view on echocardiography. (Bottom) ^99m^Tc-pyrophosphate scintigraphy showed increase myocardial uptake compared to contralateral chest with a ratio of 1.79, consistent with transthyretin cardiac amyloidosis

With scintigraphy or SPECT, bone tracers can be used to differentiate ATTR and AL cardiac amyloidosis, which have vastly different treatment strategies and survival rates. In the United States, ^99m^Tc-pyrophoscate (PYP) and ^99m^Tc-hydroxy-methylene diphosphonate (HMDP) are used off-label to diagnose ATTR cardiac amyloidosis, while ^99m^TC-3,3-diphsphono-1,2-pro-panedicarboxylic acid (DPD) is only available in Europe [78, 79]. The mechanisms of the radiotracers remain unclear but is potentially related to the binding of microcalcifications seen in ATTR deposits [80]. The myocardial uptake of radiotracers can be visually scored by comparing it to the uptake in the ribs (grade 0-absent uptake, grade 3-uptake greater than bone) or quantitatively measured as the ratio of the heart to contralateral chest uptake for PYP (Fig. 4). This ratio is not recommended for HMDP due to significantly more background noise that confound the results [79]. For PYP scintigraphy, both methods are comparable with the quantitative ratio having a sensitivity of 97% and specificity of 100% based on a ratio ≥ 1.5 [81]. In a recent study by Delbarre et al. [82], they trained a deep learning model with routine whole-body bone scintigraphy planar images to identify positive studies (visual grade ≥ 2) and achieved an accuracy of 99% on internal and external validation. This offers a potential means for screening patients for ATTR cardiac amyloidosis when undergoing bone scintigraphy for unrelated oncologic or musculoskeletal indications. More recently, it has been recognized that the use of SPECT imaging, especially when combined with CT to localize the location of PYP uptake can significantly improve the diagnostic accuracy of PYP imaging for the detection of ATTR cardiomyopathy [83].

### Metabolic Imaging

**Fig. 5:**
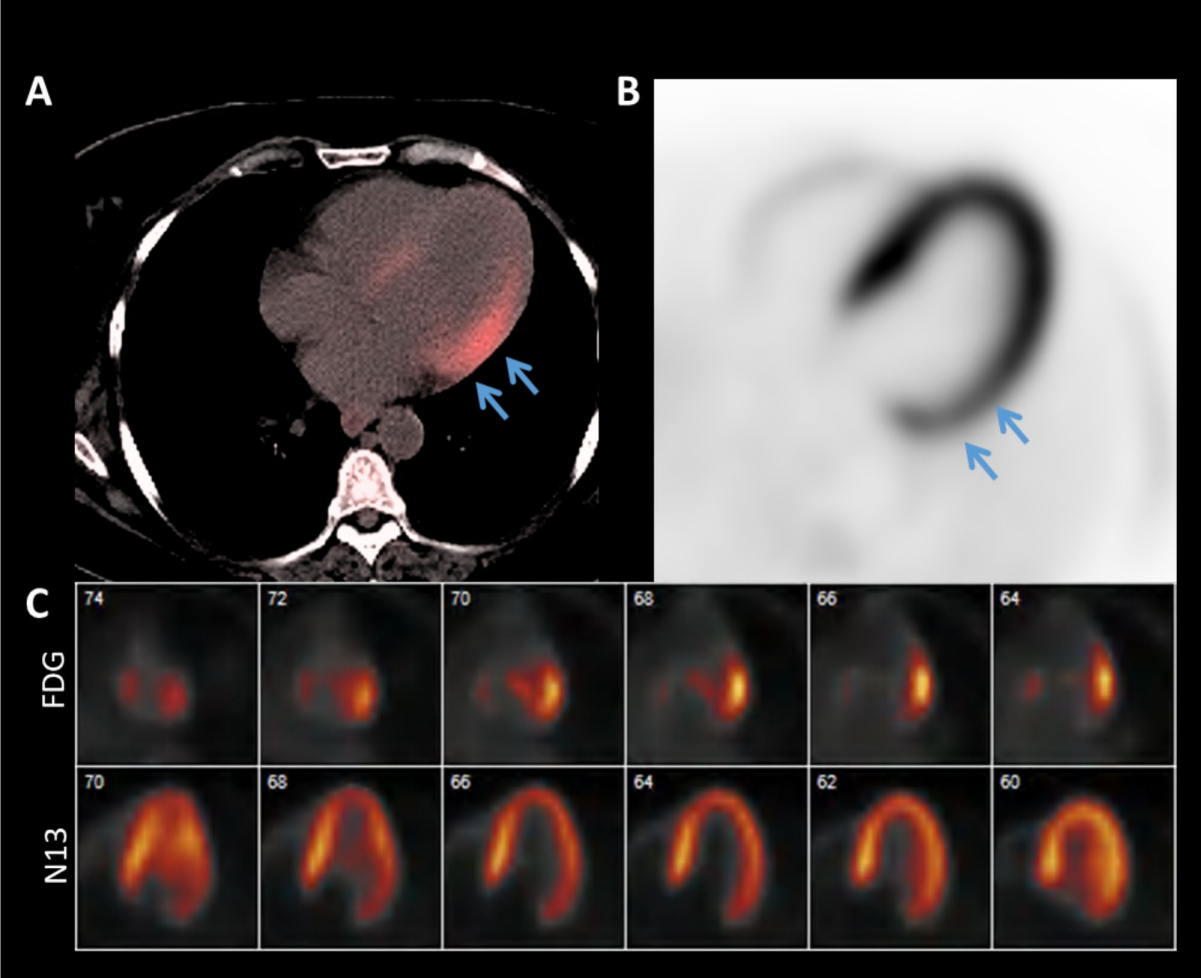
^18^F-fluorodeoxyglucose Positron Emission Tomography in Active Cardiac Sarcoidosis. An example of mismatched pattern in active sarcoidosis with ^18^F-fluorodeoxyglucose (FDG) uptake (**A**) and ^13^N-ammonia (N13)perfusion defect (**B**) in the basal to mid lateral wall (*blue arrows*). A horizontal long axis side by side comparison (**C**) of FDG uptake and N13 perfusion. From left to right, the views go from posterior to anterior

Active cardiac sarcoidosis refers to the episodes of granulomatous inflammation that lead to myocardial injury and eventual fibrosis. ^18^F-fluorodeoxyglucose (FDG) PET can detect the active phase, thus allowing for the diagnosis of cardiac sarcoidosis and treatment with immunosuppression. FDG is a glucose analog that targets activated macrophages in the granulomas, which have elevated metabolic rates. Patients must undergo either a fasting or dietary modification protocol in order to suppress consumption of glucose by healthy myocytes and promote the use free fatty acids for energy. The diet consists of the consumption of a high-fat and low-carbohydrate meals [84]. About one fourth of patients fail to successfully achieve FDG myocardial suppression due to incomplete dietary preparation [85], resulting in diffuse homogenous uptake in the myocardium. This technical challenge has encouraged the investigation of new tracers that do not accumulate in myocytes [86]. The current cardiac sarcoid PET protocols also incorporate perfusion imaging to identify microvascular dysfunction or scar in the absences of any known coronary disease. Early cardiac sarcoidosis will show focal uptake of FDG with normal perfusion, suggesting the presence of only inflammation without any scarring. A “mismatch” pattern refers to the focal areas of both FDG uptake and reduced perfusion, which represents active inflammation with myocardial injury (Fig. 5). In late stages of cardiac sarcoidosis, there may be only reduced perfusion without FDG uptake due to burned-out granulomatous tissue and scar formation [87]. A meta-analysis with 33 studies comparing the diagnostic performance of FDG PET and CMR demonstrated sensitivities of 84% and 95% and specificities of 82% and 85% respectively [88]. A few recent studies suggest using hybrid imaging with CMR and FDG PET to better classify active inflammation vs extensive scar [89, 90].

FDG PET also plays an important role in guiding the use of immunosuppression and monitoring response. A study from the Granulomatous Myocarditis Registry found that quantitative FDG uptake and LVEF > 40% was a predictor of complete response to immunosuppression with area under the curve (AUC) of 0.85 [91]. Complete response was defined as improvement in New York Heart Association functional class, freedom from ventricular arrhythmia and HF admission, and improvement of LVEF of ≥ 10%. Interestingly, none of the patients who demonstrated complete response to immunosuppression had residual FDG uptake on subsequent PET scans. However, residual FDG uptake was present in 58% of partial responders and 91% of non-responders, suggesting that PET can assess disease progression and treatment response [91].

FDG PET can also be used as alternative assessment for inflammatory cardiomyopathies including myocarditis. Although FDG PET is not routinely used for myocarditis, it can be advantageous in patients with irregular heart rates or ICDs, which contribute to imaging artifacts on CMR. In fact, parametric mapping values are significantly less reliable due cardiac implantable electronic devices [92]. When compared to endomyocardial biopsy, FDG PET had a modest sensitivity and specificity of 75% and 67% respectively [93]. There have also been a few cases reports demonstrating the use FDG PET combined with either CMR or CCT to further characterize myocarditis [94].

## Cardiac Computed Tomography

Advances in CCT has led to increases in availability, improvements in spatial resolution, and reduction in radiation and contrast doses. Because CCT are isotropic 3D acquisitions, as opposed to 2D acquisitions with CMR, views can be manipulated into any cardiac plane. Furthermore, electrocardiogram (ECG) gating allows for multiphase imaging to assess function and valve motion. When compared to CMR, CCT demonstrates excellent agreement in LV volumes and ejection fraction [95]. CCT is also useful for evaluating for thrombus when there is concern for cardio-embolic sources [96]. While coronary evaluation remains the most common indication for CCT, there is increasing emphasis on providing comprehensive training in non-coronary applications, such as structural heart disease and peri-procedural uses [97]. Below are a few non-coronary examples that frequently apply to patients with underlying cardiomyopathy.

### Electrophysiology Planning

**Fig. 6:**
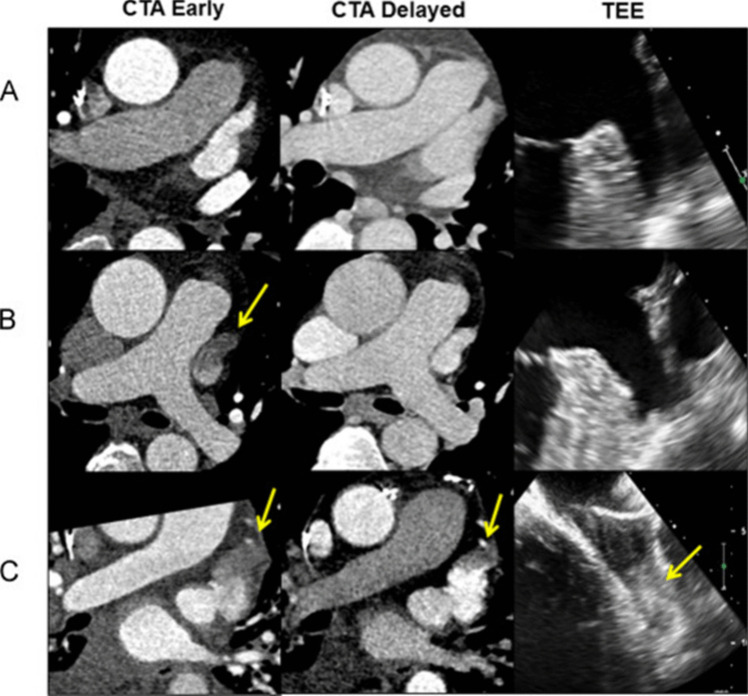
Cardiac Computed Tomography and Transesophageal Echocardiogram for Evaluation of Left Atrial Appendage Thrombus.Three examples of left atrial appendage thrombus evaluations by early and delayed computed tomography angiography (CTA) and transesophageal echocardiogram (TEE). Normal early and delayed filling of left atrial appendage (**A**) is a negative finding for thrombus. A left atrial appendage filling defect (*yellow arrows*) is also negative if it resolves on delayed imaging (**B**), otherwise it is positive if the defect has a typical appearance of a thrombus (**C**). Adapted from Bilchick et al. Heart Rhythm, 2016. 13(1):12–9, with permission from Elsevier [100]

Since the integration of CCT with electroanatomic mapping software, cross-sectional imaging has become a necessity for many electrophysiology procedures. In newly diagnosed atrial fibrillation (AF), HF is the most common complication and cause of death in patients globally [98]. Pulmonary vein isolation (PVI) is a treatment option for AF when anti-arrhythmic medication therapy fails. Prior to PVI, CCT can be used to define pulmonary vein anatomy and identify variants such as common ostium or accessory veins. CCT guided pre-procedural planning allows for optimal selection of catheter sizes and ablation approaches [99]. Additionally, CCT can assess for post-operative complications such as pulmonary vein stenosis and atrio-esophageal fistula. CCT with delayed imaging is an effective modality for excluding left atrial appendage (LAA) thrombus prior to ablation [100, 101], with a sensitivity and negative predictive value of 100% (Fig. 6). This is an attractive option for patients undergoing cardioversion or evaluation for cryptogenic stroke but are not suitable candidates for transesophageal echocardiogram (TEE) [101]. Expert consensus also recommends either preprocedural CCT or TEE to assess the LAA morphology and exclude thrombus prior to implantation of occlusion devices including the Amplatzer Amulet and the Watchman Device [102]. In a subanalysis of the SWISS APERO trial, operators that were unblinded to the pre-procedural CCT had lower radiation exposure, contrast doses, major procedure-related complications, and residual peri-device leaks [103]. CCT has also been used to identify scar with delayed enhancement imaging for ventricular tachycardia ablation [104] and define coronary sinus anatomy for cardiac resynchronization therapy [105].

### Advanced Heart Failure Therapies

With increasing life expectancy following heart transplant or left ventricular assist device (LVAD) implantation, CCT has seen an expanded role in evaluating for complications. Coronary computed tomography angiography (CCTA) offers a reliable non-invasive alternative to coronary angiography for early detection of cardiac allograft vasculopathy (CAV). The prevalence of CAV is 20% at 3 years following transplant with a 10% mortality rate after diagnosis [106]. CAV is characterized as the intimal hyperplasia and diffuse concentric luminal narrowing that leads to eventual graft failure. Patients with CAV often have minimal or non-specific symptoms due to denervation of the transplanted heart, eventually presenting with overt HF [107]. According to meta-analysis by Wever-Pinzon et al. [108], CCTA can detect CAV with stenosis ≥ 50% by invasive angiography with high sensitivity and specificity of 94% and 92% respectively. Additionally, 64-slice coronary CTA could detect intimal thickening > 0.5 mm by intravascular ultrasound (IVUS) with sensitivity and specificity of 81% and 75% respectively [108].

**Fig. 7:**
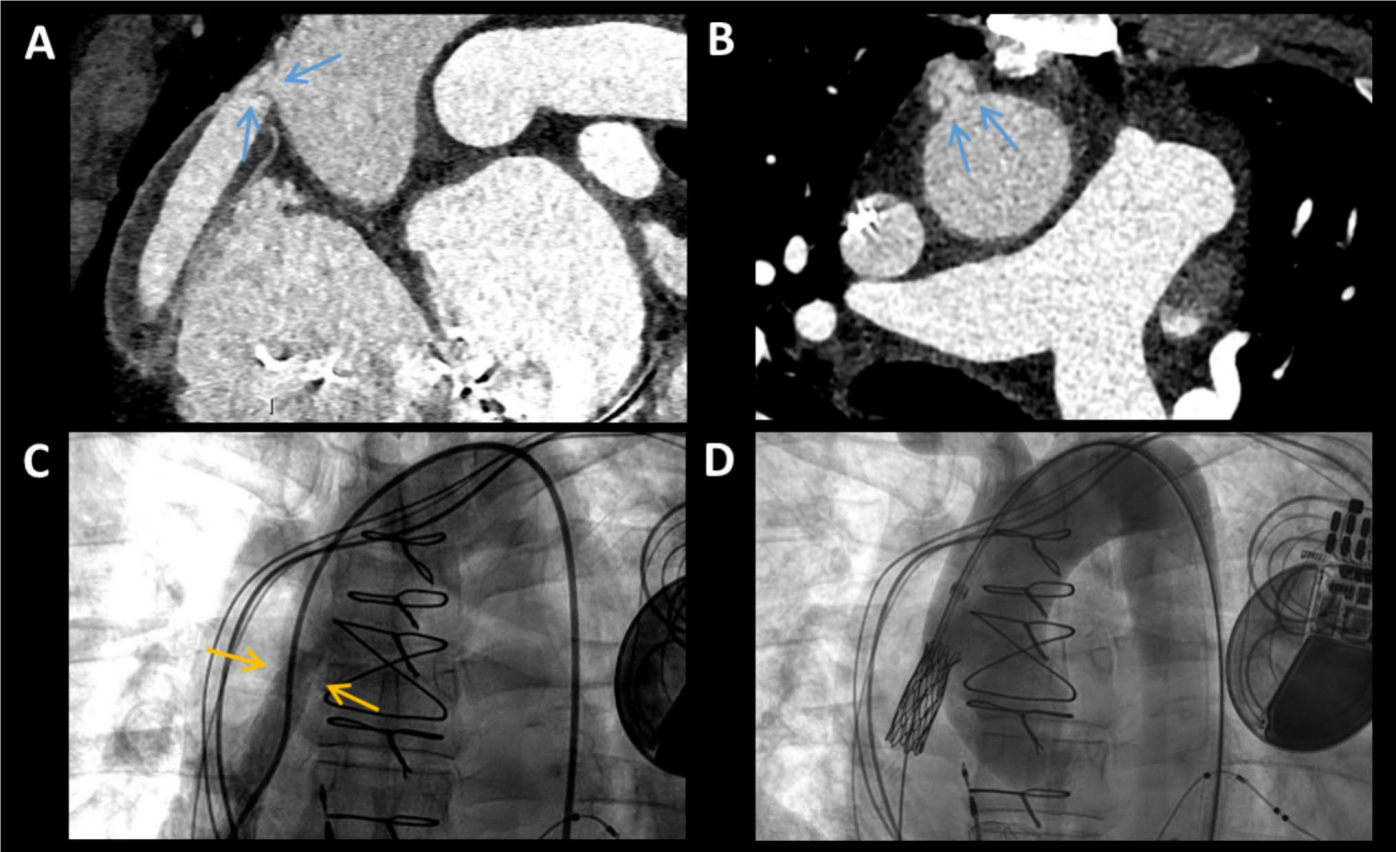
Obstruction of the Outflow Graft of Left Ventricular Assist Device. Cardiac computed tomography (CCT) in a longitudinal (**A**) and axial (**B**) plane shows a weblike thrombus *(blue arrows)* in the outflow graft of a left ventricular assist device (LVAD). Invasive angiography (**C**) demonstrated narrowing (*yellow arrows*) at the level of obstruction and the patient underwent stenting (**D**)

With the limited availability of donor hearts, there is growing use of LVAD as destination therapy for end-stage HF. Pump thrombosis and graft obstruction are common mechanical complications of LVAD. Low attenuation filling defects on CCT can represent thrombus in the pump, inflow cannula, or outflow graft (Fig. 7) [109]. In a study with 24 patients with suspected LVAD thrombosis [110], CCT demonstrated a high specificity of 100% for surgically confirmed thrombosis. However, the sensitivity was low given that most thrombi are found in the pump motor, which is not well visualized on any modality [110]. In addition, multiphase CCT can identify dynamic motion of cardiac structures that obstruct the inflow cannula and twisting of outflow graft that results in kinking of the lumen. When CCT is added to echocardiography, the diagnostic accuracy for cardio-mechanical complications of LVAD increases from 41 to 73% [111]. CCT, however, can be limited by metal artifact generated by implanted device, which can obstruct the view of the structures in question. Metal artifact reduction techniques are being developed to reduce beam hardening [112, 113]. FDG PET with CCT is another effective multimodal test that combines metabolic and anatomic findings in patients with suspected device infections including LVADs, prosthetic valves, and cardiac implantable electronic devices [114].

## Perfusion Assessment

In the initial evaluation of undifferentiated cardiomyopathy, each modality offers distinct advantages with ischemic testing. CMR, SPECT, and PET provide functional stress testing, which is useful in patients with suspected NICM or mixed cardiomyopathy but have known coronary atherosclerosis. Additionally, quantitative myocardial blood flow assessments made using CMR and PET can diagnose microvascular dysfunction, which can contribute to symptoms and be seen in NICM such as dilated cardiomyopathy, HCM, Fabry’s disease, and cardiac amyloidosis [115]. CMR and PET can also assess viability if revascularization is being considered. CCTA provides anatomic evaluation to exclude obstructive coronary artery disease in newly diagnosed cardiomyopathy [116]. Additionally, calcium scoring can identify patients with NICM who have increased risk of subsequent cardiovascular events and would benefit from statin and aspirin therapy for primary prevention [117]. Pre-procedural CCT for valve surgery can also be used to rule out obstructive proximal coronary disease, thus avoiding invasive coronary angiography [118]. There have also been significant advances in dynamic CCT myocardial perfusion imaging for improving diagnostic accuracy and quantifying myocardial blood flow [119].

## Conclusion

There is an ever-growing toolbox of imaging modalities for assessing cardiomyopathies. This review attempts to highlight ways in which CMR, CCT, nuclear imaging can provide complementary information to clinical and echocardiography findings in different disease groups. Providers should feel more comfortable and empowered to order advance cardiac imaging to diagnose rare cardiomyopathies, risk stratify patients, and guide treatment plans.

## Data Availability

No datasets were generated or analysed during the current study.
